# Medical Malpractice and Diagnostic Errors in Japanese Emergency Departments

**DOI:** 10.5811/westjem.2022.11.55738

**Published:** 2023-02-20

**Authors:** Taiju Miyagami, Takashi Watari, Taku Harada, Toshio Naito

**Affiliations:** * Juntendo University, Department of General Medicine, Bunkyō, Tokyo, Japan; † Shimane University Hospital, General Medicine Center, Department of General Medicine, Izumo City, Shimane, Japan; ‡ University of Michigan Medical School, Department of Medicine, Ann Arbor, Michigan, United States of America; § Nerima Hikarigaoka Hospital, Division of General Medicine, Tokyo, Japan; || Dokkyo Medical University Hospital, Department of Diagnostic and Generalist Medicine, Mibu, Shimotsuga, Tochigi, Japan

## Abstract

**Introduction:**

Emergency departments (ED) are unpredictable and prone to diagnostic errors. In addition, non-emergency specialists often provide emergency care in Japan due to a lack of certified emergency specialists, making diagnostic errors and associated medical malpractice more likely. While several studies have investigated the medical malpractice related to diagnostic errors in EDs, only a few have focused on the conditions in Japan. This study examines diagnostic error-related medical malpractice lawsuits in Japanese EDs to understand how various factors contribute to diagnostic errors.

**Methods:**

We retrospectively examined data on medical lawsuits from 1961–2017 to identify types of diagnostic errors and initial and final diagnoses from non-trauma and trauma cases.

**Results:**

We evaluated 108 cases, of which 74 (68.5%) were diagnostic error cases. Twenty-eight of the diagnostic errors were trauma-related (37.8%). In 86.5% of these diagnostic error cases, the relevant errors were categorized as either missed or diagnosed incorrectly; the others were attributable to diagnostic delay. Cognitive factors (including faulty perception, cognitive biases, and failed heuristics) were associated with 91.7% of errors. Intracranial hemorrhage was the most common final diagnosis of trauma-related errors (42.9%), and the most common initial diagnoses of non-trauma-related errors were upper respiratory tract infection (21.7%), non-bleeding digestive tract disease (15.2%), and primary headache (10.9%).

**Conclusion:**

In this study, the first to examine medical malpractice errors in Japanese EDs, we found that such claims are often developed from initial diagnoses of common diseases, such as upper respiratory tract infection, non-hemorrhagic gastrointestinal diseases, and headaches.

## INTRODUCTION

Diagnostic errors may occur in approximately 5% of cases in initial outpatient settings,^1^ while the error rate is 12% in emergency department (ED) settings.^2^ Studies have suggested that all patients encounter at least one diagnostic error in their lifetime.^3^ The ED environment is generally considered to create high-stress levels and is associated with high rates of medical staff sick leave and turnover, burnout, and early retirement globally.^4^,^5^ High-stress environments are also more likely to result in patient safety incidents.^6^

Japan’s emergency care system differs from that of other countries: As of 2017, the number of EDs in Japan (approximately 4,000) and the number of certified emergency specialists (approximately 4,500) are almost equal, meaning that there are few specialists in each ED.^7^,^8^ Emergency physicians are required to train for at least three years at an accredited facility recognized by the Board of Emergency Medicine Moreover, those who have experience in situations such as cardiopulmonary resuscitation and focused assessment with sonography for trauma cases, as well as 20 emergency diseases such as cardiopulmonary arrest and shock, and have passed a written examination, become a specialist.^9^ Additionally, there are not many people in Japan who claim to be solely emergency physicians.

Since the board certification system for emergency physicians was officially launched in 2007, the number of applicants has remained at 300–400 per year.^10^ Therefore, care in the ED is often provided by non-emergency specialists (such as other physicians or surgeons, depending on each hospital policy, alongside their regular duties) in high-stress settings, creating an environment that might be prone to diagnostic errors and many medical malpractice lawsuits. In other countries, there have been several investigations of medical malpractice in the ED, suggesting that diagnostic errors and procedural problems contribute to malpractice.^11^–^13^ However, few studies have examined diagnostic error-related malpractice in Japanese EDs.

While in this study we used data from the largest legal database in Japan, the resulting number of cases is quite small compared to the number of such cases that occur in the United States (US). According to the Japanese Supreme Court report, approximately 300–700 medical lawsuits are adjudicated each year in Japan, including those heard in brief and district courts,^14^ meaning that Japan has only about 5% of the number of medical malpractice cases as the US.^15^ The purpose of this study was to identify error-prone initial and final diagnoses using data from medical malpractice lawsuits related to diagnostic errors that occurred in Japanese EDs and to create awareness among working emergency specialists.

## MATERIALS AND METHODS

### Study Design

For this study, we collected cases from the largest database of litigation in Japan (Westlaw Japan K.K.).^14^ The database contains information on more than 200,000 lawsuits of all types, from which we extracted data on medical lawsuits from 1961–2017. All litigation data in the database were anonymized, but all the medical information data used in this study could be extracted. In Japan, unlike in the US and the United Kingdom, the jury system was implemented between 1923–1943 and resumed in 2009.^16^ As a result, for most of our history, trials by jury have not been held and were instead conducted by certified judges.

**Population Health Research Capsule**

What do we already know about this issue?

*In Japan non-emergency specialists often provide emergency care, which frequently leads to diagnostic errors and associated medical malpractice*.

What was the research question?

*We examined diagnostic error-related medical malpractice lawsuits that involved Japanese emergency departments (ED)*.

What was the major finding of the study?

*We evaluated 108 cases, of which 74 (68.5%) were related to diagnostic errors*.

How does this improve population health?

*Awareness of the frequency of diagnostic errors in the ED and initial diagnosis can help reduce future errors*.

### Setting and Participants

Permutational combinations of “medical claims,” “medical malpractice,” “medical lawsuits,” “diagnostic errors,” “misdiagnosis,” and “delayed diagnosis” were used as keywords related to claims. We combined all claim cases into a single tabular list (3,430 cases). Before extracting the data, the corresponding author and a senior medical student licensed to practice law established exclusion criteria, namely 1) duplicate cases, 2) intentional crimes, 3) robberies, 4) money disputes, and 5) veterinary claims.

We excluded 751 duplicate cases, 707 cases that met the exclusion criteria, 34 cases that constituted an “unfair suit” (defined as a claim that a lawyer decides is unreasonable), 136 cases with a non-physician defendant, and 1,693 cases that were not related to the ED (Figure).

### Ethics

This study is based on data that has already been published as legal proceedings and is part of the public record; thus, patient consent was not required. Institutional review board approval was not required and was waived by the university hospital.

### Variables

The data used in this study included patient background (age, gender, treatment outcome, initial diagnosis, final diagnosis, and whether the case was trauma-related); physician characteristics (department and clinical setting); and litigation details (duration, sequelae, medical outcomes, judgment, and billing indemnity amount). Among litigation details, the term “ judgment “ is defined as the judgment of a court of law. Additionally, the term “ billing amount “ is the amount the patient’s attorney requested prior to trial. The term “ indemnity paid “ is defined as the amount ordered to be paid by a court judgment. Doctor specialty classification was based on the Japanese medical specialty board (2019).^17^ All of the targeted cases were labeled as diagnostic error-related claims (DERC) or non-DERC by the three university students and confirmed by the three co-authors.

The papers were first evaluated by two people in a blind and independent environment, of which seven cases (93.5% concordance rate) were discordant between the two evaluators, and a third person made the final evaluation of the discordant cases. The evaluating authors are all general medicine doctors trained at medium-to-large hospitals in Japan, which are the only facilities of the 552 total hospitals in the country where emergency medicine specialization can be obtained.^9^ In these hospitals, general medicine is the main specialty, but EDs handle over 5,000 emergency cases per year. These hospitals see emergencies far more than the other hospitals in Japan, as 45.1% of hospitals with EDs receive fewer than 360 ambulances per year.^18^ Moreover, one of the authors still works in the ED and teaches residents. As mentioned above, this type of department has led to many generalists working as emergency physicians.

To minimize bias during the case review, we used common definitions of diagnostic error: “delay in diagnosis” and “missed or wrong diagnoses.”^19^ The final diagnosis of non-trauma cases was confirmed by analyzing the database, while case classifications were determined through consultation with the two authors who are general medicine doctors with several years of experience working in the ED. The cases were categorized into four categories. The first three (infection, tumor, and vascular disease) account for about 74% of medical claims due to diagnostic errors in the US and are known as the “Big 3”; other cases were combined into a fourth category.^20^

Judgments were decided final if made by the Supreme Court, high courts, or local district courts.

### Main Outcomes

The main outcomes for the study were the type of diagnostic error, the final diagnosis assessed from the initial diagnosis of non-trauma cases, and the diagnostic classification of trauma cases.

### Data Analysis

All payment values were adjusted to the 2017 equivalent using the Japanese Consumer Price Index (available at https://www.stat.go.jp/data/cpi/, Japanese Ministry of Internal Affairs and Communications). We converted each payment amount from Japanese yen to US dollars (¥110 = $1 on March 20, 2020). Continuous variables are presented as median values, and interquartile ranges (IQR); categorical variables are presented as numbers and proportions (%) of the corresponding cases. We used JMP PRO software version 13.0 (SAS Institute, Cary, NC,) for all calculations.

## RESULTS

We evaluated a total of 108 cases in this study; 74 (68.5%) of the cases were related to diagnostic errors. Twenty-eight of the diagnostic errors were trauma-related (37.8%) (Figure). The frequency of diagnostic errors of all types (missed or wrong diagnoses and diagnostic delay) within the ED leading to medical malpractice lawsuits was 68.5%. The mean age of the patients was 32 years (IQR 16–54), and 66.7% were men. The median claim amount was $443,155 (IQR $232,295–$689,239), 42.6% of the cases ended with a judgment in the favor of the plaintiff, and the median amount of the judgment was $30,393 (IQR $0–$291,593). The median duration of litigation was six years, with a mortality rate (the patient died before receiving a judgment) of 79.6%; the median claim amount of diagnostic error cases was $449,759 (IQR $227,199–$684,875); 59.5% of the cases ended with a judgment in favor of the plaintiff of diagnostic error cases; and the median amount acceptance of diagnostic error cases was $224,121 (IQR $53,106–$388,336).

Error types consisted of missed or wrong diagnosis in 86.5% of cases and diagnostic delay in 13.5% (Table 1).The departments involved in examining the cases of diagnostic error in the ED were the following: internal medicine in 27 cases (36.5%); surgery in 24 cases (32.4%); pediatrics in seven cases (9.5%); and EDs in only three cases (4.1%). Intracranial hemorrhage was the most common final diagnosis of trauma-related errors in 12 cases (42.9%), followed by digestive system disease in 10 cases (35.7%), and pulmonary system disease in four cases (14.3%). Traffic injury was the most common trauma-related diagnosis in 15 cases (53.6%), and four of the five alcohol-related cases had a final diagnosis of intracranial hemorrhage (Table 2).

The final diagnoses of non-trauma-related diagnostic errors were related to the vascular system in 18 cases (39.1%), infection in 16 (34.8%), and other in 12 (26.1%); no cases were tumor-related. The most common vascular diseases were acute myocardial infarction and subarachnoid hemorrhage, with five cases each. The most common infections were epiglottitis and meningitis, with four cases each (Table 3).

The most common initial diagnoses of non-trauma-related errors were upper respiratory tract infection (10 cases, 21.7%), non-bleeding digestive tract disease (seven cases, 15.2%), and primary headache (five cases, 10.9%). When the initial diagnosis of upper respiratory tract infection was made, the most common final diagnosis was epiglottitis (four cases, 40%). When the initial diagnosis of non-bleeding digestive tract disease was made, the most common final diagnosis was peritonitis (three cases, 42.9%). When the initial diagnosis of primary headache was made, the most common final diagnosis was subarachnoid hemorrhage (three cases, 60.0%) (Table 4).

## DISCUSSION

In this study we analyzed 108 medical lawsuits in Japanese EDs and confirmed that 68.5% were due to diagnostic errors. Of these, we examined in detail 74 medical malpractice cases due to diagnostic errors in the ED. The settlement rate was 59.5%, and the amount accepted was $224,121 (IQR $53,106–$388,336). The mortality rate was 82.4%. The settlement rate was 59.5%, and the amount accepted was $224,121 (IQR $53,106–$388,336). The mortality rate was 82.4%.

The most common trauma-related final diagnosis was intracranial hemorrhage, while the most common non-trauma-related final diagnosis was associated with the vascular system. The most common initial diagnoses were upper respiratory tract infection, non-bleeding digestive tract disease, and primary headache. For some of these cases, the initial diagnoses were in different disease group categories than the final diagnoses. To make the results of the survey in Japan easier for readers to understand, we will focus our discussion on the following five points: 1) background of medical litigation and diagnostic errors; 2) differential diagnoses prone to diagnostic errors; 3) trauma-related errors; 4) initial diagnosis with particular attention to non-traumatic diagnostic errors; and 5) future prevention and countermeasures.

### Background of Medical Litigation and Diagnostic Errors

Several previous studies reported that the judgment for the plaintiff rate was 13.3% in the ED setting for medical malpractice in Taiwan^21^ and 31% in 2020 in the US.^11^ Diagnostic errors were involved in 35–37% of the medical lawsuits in the ED in the US.^22^,^23^ This is considerably lower than the rate in Japan (68.5%). In the US, the emergency physician was the most common specialist who made errors in the ED (19%), followed by internists, family physicians, orthopedic surgeons, and general surgeons.^23^ The differences between the present study and others may be due to the differences in trials and culture in each country, and the fact that only relatively serious cases are brought to trial due to the small number of medical lawsuits in Japan (approximately 1/21 of those in the US) as a fundamental background.^15^

In the present study, there were many vascular final diagnoses and no tumor-related errors. This may have been influenced by the differences in ED systems and insurance systems between Japan and the US. Japan’s emergency call system allows patients to call an ambulance for free, and there are no rules such as the Emergency Medical Treatment and Active Labor Act.^8^ Therefore, emergency physicians can refuse ambulances at their discretion. It is common for patients to be rejected by multiple hospitals after boarding an ambulance. Although the rate of diversion from one hospital to another is not published, according to the 2020 data, it took an average of 30 minutes from emergency medical service arrival at the scene to an accepting hospital arrival.^24^ As the emergency medical team attempts to find a hospital, the patient’s vital signs are likely to collapse, as cardiovascular disease and other time-sensitive conditions may worsen. Consequently, the emergency physician may make further diagnostic or treatment errors, due to lack of specialty expertise that warrants the transfer.

### Differential Diagnoses Prone to Diagnostic Errors

A previous study of diagnostic errors in the ED showed that the top three results in the US were vascular (39.6%), infection (21.2%), and tumor (7.9%).^20^ According to three studies of US medical lawsuits, the most common final diagnoses related to diagnostic errors in the ED are acute myocardial infarction, appendicitis, pulmonary embolism, and fractures.^11^,^22^,^23^

### Trauma-related Errors

As for trauma-related errors, data from previous studies that only evaluated trauma-related cases are scarce and not comparable. However, the findings of this study suggest that when patients reach a hospital with alcohol-related trauma, more attention should be paid to the presence of a latent intracranial hemorrhage (with errors in 33% of cases).

### Initial Diagnosis with Particular Attention to Non-traumatic Diagnostic Errors

In this study the most common initial erroneous diagnoses in non-trauma-related diagnostic errors were upper respiratory tract infection, non-bleeding digestive tract disease, and primary headache. Previous studies have reported low concordance rates for the initial diagnosis of upper respiratory tract infections in the ED.^25^ In connection with the results of this study, we need to consider the possibility that patients presenting with upper airway symptoms in the ED may have a different initial diagnosis. Gastroenteritis is often given as an initial diagnosis of patients who ultimately are diagnosed with cerebellar hemorrhage in the ED and primary care,^26^ and even in cases where gastrointestinal disease is suspected, it is important to conduct a detailed history and physical examination because it may not be of gastrointestinal symptoms.^27^,^28^

For primary headache, it was reported that 36% of subarachnoid hemorrhage cases were diagnosed with primary headache, such as migraine or muscle tension headache, at the first visit.^29^ Of those diagnosed with tension-type headache, 50.2% had a different final diagnosis, and 30.3% of those patients were diagnosed with secondary headache.^30^ Another previous study found that the most common diagnostic error in patients discharged with nonspecific headache was ischemic stroke (18%).^31^ Previous studies have pointed out that it is important to be aware of the “red flag” signs of headache (new onset in patients over 50 years old with impaired consciousness, thunderclap headache, worst headache ever experienced, altered mental status, nausea/vomiting, focal neurological deficits, etc).^32^

### Future Prevention and Potential Strategy

A previous study has shown that physicians who have faced medical malpractice lawsuits gravitate toward “defensive medicine” such as excessively ordering tests, performing diagnostic procedures, and referring patients for consultation and that they become “more conservative” such as avoiding trauma surgery and patients who suffer from complex medical problems.^33^ So we should consider how to reduce the number of medical malpractice occurrences caused by diagnostic errors in the ED setting. For example, a previous study reported that outpatient follow-up after an ED visit reduces patient mortality,^34^ and that improving teamwork, patient engagement, and learning from diagnostic errors are also effective methods.^35^ Other reports suggest that failure to assess, communicate, and respond to ongoing symptoms is a common error made by clinicians in the ED and that more attention is needed.^36^ Understanding and addressing error-prone situations in this way will help reduce errors.

It is also important to reconsider a diagnosis when a differential diagnosis does not match the symptoms, signs, or tests and to consider the possibility of uncommon or common atypical cases after ruling out common diseases to reduce errors.^37^ Therefore, the initial and final diagnosis figures that led to the lawsuits in this study could be used as part of a checklist to reduce errors in the ED, which could lead to fewer errors in the future. The use of cognitive forcing tools by clinicians in busy settings such as EDs has been reported to have a positive subjective impact on diagnostic accuracy and thoughtfulness.^38^ In the Netherlands, the number of patients coming to the ED has increased since the number of emergency specialists has increased; however, the number of medical malpractice suits has decreased.^12^ In Japan, the number of emergency specialists has increased threefold between 2004–2017,^7^ and the trend of diagnostic errors in the ED is likely to change. This will need to be assessed with further research. However, we think it is important to increase the number of emergency specialists.

## LIMITATIONS

This study has several limitations. First, while we used the largest database of medical malpractice in Japan, it does not cover all medical claims nor does the database include out-of-court settlements. Therefore, it is unclear to what extent settlements occurred prior to medical malpractice. In addition, the information was based on a database of medical lawsuits, and it was difficult to analyze confounding factors in the social environment, changes in the legal system, or the trends of the forms of claims with the development of technology in medicine. Second, it is unclear from this database to what extent diagnostic errors in Japan lead to medical malpractice claims, as there is no actual data on existing diagnostic errors. Third, the database is anonymized trial data, which means that the personal information of the medical personnel in charge cannot be extracted, making it less than ideal for research on diagnostic errors. Fourth, as the database is based on Japan’s judicial administrative system, it is difficult to make simple comparisons with other countries in terms of the amount and rate of medical malpractice occurrence.

Finally, the system of emergency care in Japan is very different from that in other countries; thus, a simple comparison may be difficult in this respect as well. Despite these limitations, to the best of our knowledge, this is the first and largest study to investigate medical malpractice related to diagnostic errors in Japanese EDs; as such, it could influence future efforts to improve patient safety in EDs.

## CONCLUSION

Of the 108 malpractice claim cases we analyzed that occurred in Japanese EDs, we identified that 68.5% of the cases were due to diagnostic errors. Specifically, relatively common conditions at the initial visit, such as upper respiratory tract infection, non-hemorrhagic gastrointestinal diseases, and primary headache diagnosis, were serious illnesses and resulted in medical litigation, which stood out in our extracted claims cases. The emergency care setting is demanding and challenging for physicians; future research is needed to determine the true causes and the strategies that should be used to prevent diagnostic errors.

## Figures and Tables

**Figure:**
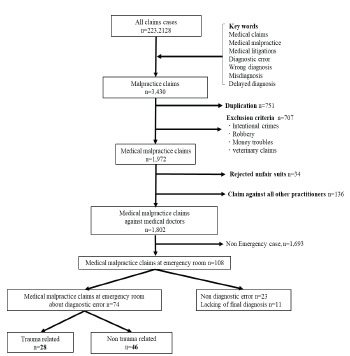
Participant flowchart. Medical lawsuits in Japan from 1961–2017.* *From the total number of lawsuits, “medical claims,” “medical malpractice,” “medical litigation,” “diagnostic error,” “wrong diagnosis,” “misdiagnosis,” and “delayed diagnosis” were used as keywords to identify cases. Exclusion criteria and unfair suits were defined as claims that a lawyer decides are unreasonable.

**Table 1:** Findings of emergency department medical malpractice study in Japan 1961–2017.*

Characteristics	Median (IQR) or number (%) N=108
Patient age (IQR)	32 (16–54)
Male gender, number (%)	72 (66.7)
Adjusted total billing amount ($)	443,155 (232,295–689,239)
Claims with final judgment resulting in payment (%)	46 (42.6)
Adjusted median indemnity paid amount ($)	30,393 (0–291,593)
Duration of claim (years)	6 (5–7)
Outcome	
Deaths (%)	86 (79.6)
Sequelae (%)	20 (18.5)
Full recovery (%)	2 (1.9)
Cases of diagnostic error (%)	74 (68.5)

Characteristics of diagnostic error cases	Median (IQR) or number (%) N= 74

Adjusted total billing amount of diagnostic error cases ($)	449,759 (227,199–684,875)
Claims with a final judgment resulting in payment of diagnostic error cases (%)	44 (59.5)
Adjusted median indemnity paid amount of diagnostic error cases ($)	224,121 (53,106–388,336)
Duration of claim of diagnostic error cases (years)	6 (5–7)
Outcome of diagnostic error cases	
Deaths (%)	61 (82.4)
Sequelae (%)	10 (13.5)
Full recovery (%)	2 (2.7)
Error type	
Missed or wrong diagnosis (%)	64 (86.5)
Diagnostic delay (%)	10 (13.5)
Trauma related (%)	28 (37.8)

* This study collected data on medical malpractice lawsuits from 1961–2017.

The billing amounts and indemnity paid amounts were adjusted to the 2017 equivalent using the Japanese Consumer Price Index (shown in US dollars).

*IQR*, Interquartile range.

**Table 2:** Trauma-related diagnostic error in the emergency department (n=28): a medical malpractice study in Japan 1961–2017.*

Trauma final diagnosis	Total number	Traffic injury n, (%)	Alcohol-related n, (%)	Others n, (%)
Intracranial hemorrhage	12	4 (33.3)	4 (33.3)	4 (33.3)
Trauma bowel injury	10	6 (60)	1 (10)	3 (30)
Pulmonary	4	3 (75)	0	1 (25)
Musculoskeletal system	2	2 (100)	0	0

* Trauma-related errors were divided by disease group and categorized as traffic-related, trauma-related, alcohol-related, and others.

**Table 3:** Non-trauma related final diagnosis (n=46).*

Disease	Total n (%)
Vascular	18 (39.1)
Acute myocardial infarction	5 (10.9)
Subarachnoid hemorrhage	5 (10.9)
Aortic dissection	4 (8.7)
Infection	16 (34.8)
Epiglottitis	4 (8.7)
Meningitis	4 (8.7)
Peritonitis	3 (6.5)
Tumor	0
Others	12 (26.1)
Bronchial asthma	2 (4.3)
Acute pancreatitis	2 (4.3)
Intestinal obstruction	2 (4.3)

* Most common non-trauma categories of malpractice suits related to diagnostic error; % is percentage of total number.

**Table 4:** Top three initial diagnoses of cases of diagnostic errors in non-trauma cases and their final diagnosis.*

Initial diagnosis (total number)	Final diagnosis of 1st rank n, (%)	Final diagnosis of 2nd rank n, (%)	Final diagnosis of 3rd rank n, (%)
Upper respiratory tract infection 10	epiglottitis 4 (40)	meningitis 2 (20)	appendicitis, pneumonia, cerebral stroke, heat illness 1 (10)
Non-bleeding digestive tract disease 7	peritonitis 3 (42.9)	subarachnoid hemorrhage 2 (28.6)	intestinal obstruction, intestinal invagination (intussusception) 1 (14.3)
Primary headache 5	subarachnoid hemorrhage 3 (60)	cerebral stroke 2 (40)	

* Initial and final diagnoses of non-traumatic diagnostic error cases arranged by rank; % is percentage of the total number of each initial diagnosis.
